# Getting CD19 Into Shape: Expression of Natively Folded “Difficult-to- Express” CD19 for Staining and Stimulation of CAR-T Cells

**DOI:** 10.3389/fbioe.2020.00049

**Published:** 2020-02-07

**Authors:** Elisabeth Lobner, Anna Wachernig, Venugopal Gudipati, Patrick Mayrhofer, Benjamin Salzer, Manfred Lehner, Johannes B. Huppa, Renate Kunert

**Affiliations:** ^1^Department of Biotechnology, University of Natural Resources and Life Sciences, Vienna, Austria; ^2^Institute for Hygiene and Applied Immunology, Center for Pathophysiology, Infectiology and Immunology, Medical University of Vienna, Vienna, Austria; ^3^St. Anna Children’s Cancer Research Institute, Vienna, Austria

**Keywords:** CD19, difficult-to-express protein, BAC, chemical chaperones, T cell activation, CAR-T cell, TIRF

## Abstract

The transmembrane protein CD19 is exclusively expressed on normal and malignant B cells and therefore constitutes the target of approved CAR-T cell-based cancer immunotherapies. Current efforts to assess CAR-T cell functionality in a quantitative fashion both *in vitro* and *in vivo* are hampered by the limited availability of the properly folded recombinant extracellular domain of CD19 (CD19-ECD) considered as “difficult-to-express” (DTE) protein. Here, we successfully expressed a novel fusion construct consisting of the full-length extracellular domain of CD19 and domain 2 of human serum albumin (CD19-AD2), which was integrated into the *Rosa26* bacterial artificial chromosome vector backbone for generation of a recombinant CHO-K1 production cell line. Product titers could be further boosted using valproic acid as a chemical chaperone. Purified monomeric CD19-AD2 proved stable as shown by non-reduced SDS-PAGE and SEC-MALS measurements. Moreover, flow cytometric analysis revealed specific binding of CD19-AD2 to CD19-CAR-T cells. Finally, we demonstrate biological activity of our CD19-AD2 fusion construct as we succeeded in stimulating CD19-CAR-T cells effectively with the use of CD19-AD2-decorated planar supported lipid bilayers.

## Introduction

**FIGURE 1:**
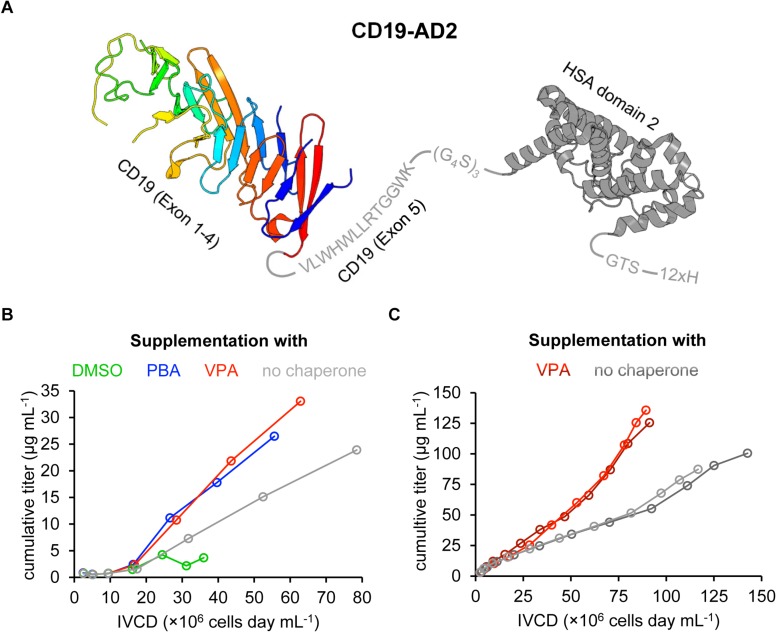
Design and recombinant expression of the CD19-AD2 fusion construct. **(A)** Schematic representation of the expressed CD19-AD2 fusion construct. The crystal structure of CD19 (PDB ID: 6AL5) encompassing exons 1–4 is depicted in rainbow colors from blue (N-terminus) to red (C-terminus) followed by exon 5 (residues V279-K291 in gray). This extracellular domain of CD19 is C-terminally fused to domain 2 of human serum albumin (HSA) (PDB ID: 6JE7), via a glycine serine linker shown in gray. The fusion tag is followed by a polyhistidine tag. **(B)** The impact of chemical chaperones on cell growth and CD19-AD2 expression between day 3 and day 9 is demonstrated as the cumulative titer versus the integral of viable cell density (IVCD). Chemical chaperones (i.e., 2% DMSO; 1 mM PBA; 0.5 mM VPA) were supplemented on days 5 and 7 in fresh medium. Titers of CD19-AD2 were determined with anti-HSA sandwich ELISA. **(C)** Cumulative titer versus IVCD comparison of CD19-AD2 product generation with addition of VPA (shades of red) and without any chemical chaperones (shades of gray) within a semi-continuous perfusion in spin bioreactor tubes. Data shown represent duplicate experiments, respectively. From day 5 on cultures (shades of red) were daily supplemented with 0.5 mM VPA. Titers were determined with a BLI assay.

Cluster of differentiation 19 (CD19) functions in B cells as the dominant signaling component alongside CD21, CD81 and CD225 (Tedder et al., 1997; Tedder, 2009; Wang et al., 2012). Since its expression is restricted to B cells, CD19 represents a perfectly suited antigen for cell type-specific immunotherapies. To date, two CD19-specific chimeric antigen receptor (CAR)-T cell therapies for the treatment of acute lymphoblastic leukemia (ALL) and diffuse large B cell lymphoma (DLBCL) have been approved (June and Sadelain, 2018). Despite its relevance as a therapeutic target, not much is known about CD19 signaling on a molecular level, which is at least in part attributable to the nature of its extracellular domain (CD19-ECD) as being particularly “difficult-to-express” (DTE). DTE proteins are typically characterized by very low expression titers and insufficient protein quality resulting from misfolding and aggregation, which precludes any detailed studies (Alves and Dobrowsky, 2017). In case of CD19-ECD this was illustrated by the formation of disulfide bonded oligomeric aggregates (Chang et al., 2018) and the fact that 40 L of cell culture supernatant were required for providing 14 mg protein to resolve the X-ray structure of an N-glycosylation mutant of CD19-ECD (Teplyakov et al., 2018). In contrast to the formerly suggested two independent Ig-like domains separated by a disulfide bonded non-Ig-like domain (Tedder and Isaacs, 1989; Zhou et al., 1991) the crystal structure of the glycan mutant of CD19-ECD revealed a novel fold in which the Ig-like domain of one Ig fold is inserted into the other as shown in Figure 1A (Teplyakov et al., 2018).

To overcome the bottlenecks associated with difficult protein expression, we combined different strategies which are known to facilitate the generation of soluble and active proteins (Papaneophytou and Kontopidis, 2014). Since fusion tags have previously been observed to reduce proteolytic degradation and improve solubility and stability of proteins by acting as a nucleus for folding, we opted to fuse CD19-ECD (Creighton, 1997; Young et al., 2012; Paraskevopoulou and Falcone, 2018) C-terminally with the domain 2 (AD2) of human serum albumin (HSA) (Figure 1A). Full-length HSA consists of three domains and has been extensively employed in fusion constructs (Muller et al., 2007; Rogers et al., 2015). Of note, when expressed as a fusion protein, AD2 has shown to be significantly more efficient with regards to productivity, compared to full-length HSA or the domain 1 of HSA (Joung et al., 2009).

Moreover, the choice of the expression vector used for the generation of a stable producer cell line is considered critical for expression of recombinant protein (Wurm, 2004). Since the integration site of conventional expression vectors into the host genome defies prediction, the expression of the protein of interest can be silenced over time by so-called chromatin positional effects. To circumvent gene silencing we took advantage of a bacterial artificial chromosome (BAC) vector, which is equipped with genetic regions that are considered as open chromatin and thus less affected by positional effects (Blaas et al., 2009, 2012; Kunert and Casanova, 2013; Mader et al., 2013).

Maturation and correct assembly of the recombinant protein within the endoplasmic reticulum (ER) is the next step to enable proficient secretion of a recombinant protein. To improve folding and circumvent aggregation or degradation through activation of the unfolded protein response (UPR) (Hetz and Papa, 2018), we supplemented chemical chaperones to the cultivation medium. These chaperones may block the UPR activation by acting directly on its signal transduction pathway and help the correct folding of aggregate-prone proteins by promoting the expression of molecular chaperones (de Almeida et al., 2007; Bandyopadhyay et al., 2012). Above all, addition of valproic acid (VPA) proved to increase product titers although it is known to cause a reduction in cell growth (Segar et al., 2017).

Here, we present the recombinant production of a fusion protein of the “difficult-to-express” protein CD19-ECD in sufficient amounts for conducting all described experiments. Furthermore, we determine the protein quality and functionality through a variety of biochemical methods. Finally, we confirm structural integrity as well as biologic activity of recombinantly expressed CD19-AD2 by effective stimulation and synapse formation of CD19-CAR-T cells confronted with supported lipid bilayers (SLBs) featuring recombinant CD19-AD2.

## Results

### Design and Recombinant Expression of the CD19-AD2 Fusion Construct

Based on the UniProtKB (ID: P15391) we designed the CD19-AD2 fusion construct to include the extracellular region of human CD19 (CD19-ECD) extending from position P20 to position K291, hence encompassing exons 1 to 4 as well as 13 residues from exon 5 (Figure 1A) (Zhou et al., 1991). To avoid misfolding and the formation of multimeric CD19-ECD and to increase in general protein solubility, domain 2 of HSA (AD2) was fused to the C-terminus of CD19-ECD via a glycine-serine linker (Figure 1A). Lastly, a 12x histidine tag was added to the C-terminus of the CD19-AD2 construct via a 3-amino acid GTS linker to serve protein purification and also protein anchoring to the Ni-NTA containing planar SLBs for later assays.

To support augmented protein expression levels the expression cassette of CD19-AD2 was cloned into the *Rosa26*^BAC^ vector for stable transfection of the CHO-K1 cell line. To further optimize expression yields different chemical chaperones were tested as a media supplement. It has been reported that addition of 2% DMSO reduces cell growth but simultaneously increases titers with significantly reduced protein aggregates (Hwang et al., 2011). In the same study, titers were also found slightly increased after addition of 1 mM 4-phenylbutyric acid (PBA) without protein aggregation being affected. Of note, aggregation had also been observed to be considerably reduced in the presence 1 mM PBA with a concomitant increase in Fc-fusion protein-yields in a transfected CHO cell line (Johari et al., 2015). Various other studies have employed VPA to further boost protein production and promote native folding of a given protein to be expressed (Backliwal et al., 2008; Wulhfard et al., 2010; Jager et al., 2013; Kuryatov et al., 2013). Segar et al. (2017) supplemented their IgG expressing CHO cell line with 0.5 mM VPA and reached a threefold increase in productivity. Because improvements in protein expression appeared to depend highly on the nature of the protein in question, we tested a selection of chemical chaperones (i.e., 2% DMSO, 1 mM PBA, and 0.5 mM VPA) within a batch process using the recombinant CD19-AD2 expressing CHO-K1 cell line (Figure 1B). During the 9-day process period, viabilities remained high and never dropped below 97% for PBA-, VPA-treated cells and cells cultured without any supplementation, whereas DMSO treatment caused a significant loss in viability. In addition, cells treated with DMSO could not exceed a density of 1 × 10^7^ cells mL^–1^ while cell growth was less affected by the addition of PBA or VPA. From day 5 on cells were incubated with chemical chaperones which caused in general a significant reduction in cell growth, whereby the highest cell density of about 3 × 10^7^ cells mL^–1^ could only be reached in the absence of a chemical chaperone. However, the highest CD19-AD2 titers with up to 33 μg mL^–1^ could be achieved by adding VPA. Data represented as CD19-AD2 titers versus the integral of viable cell density (IVCD) clearly show that treatment with VPA and PBA leads to higher productivities within a shorter time period than treatment with DMSO or the control without any supplementation (Figure 1B). Supplementation with DMSO impaired cell growth, viability as well as protein production. Taken together, these data are consistent with previously reported observations that chemical chaperones reduce growth rate while maintaining high viabilities and acting directly on the protein folding machinery and the UPR (de Almeida et al., 2007; Bandyopadhyay et al., 2012; Segar et al., 2017). Based on this preliminary experiment, supplementation with 0.5 mM VPA was chosen for the enhancement of CD19-AD2 protein expression in a semi-continuous perfusion mode. This process was performed over a time period of 13 days and allowed us to culture cells at high cell densities with high viabilities. Although, the viable cell density was on average 1.5-fold higher without addition of VPA, titers were significantly increased through addition of VPA (Figure 1C). The material used for quality control and functionality tests was purified by affinity and size exclusion chromatography and a final concentration step and resulted in a monomeric CD19-AD2 preparation (1.04 mg/ml). Importantly, this CD19-AD2 preparation resulted in high protein quality, is stable when stored at −80°C and gave rise to quantities that were high enough for conducting all of the following experiments.

### Verification of the Monomeric Fold and Functionality of CD19-AD2

Since the quality of the expressed protein determines the degree of success for all subsequent experiments, the purified CD19-AD2 was investigated with respect to aggregate formation and monomeric fold. So far, only few reports have addressed the recombinant expression of soluble CD19-ECD, mainly as a fusion protein (De Oliveira et al., 2013; Chang et al., 2018; Teplyakov et al., 2018). Furthermore, they did not perform comprehensive protein characterization. Commercially available recombinant CD19-ECD fusion proteins are typically characterized by SDS-PAGEs or Western Blot analysis showing protein samples that were reduced prior loading and thus exhibiting a single band of monomeric protein. Of note, Western blot analysis of recombinant CD19-ECD by Chang and colleagues indicated the predominant presence of oligomeric forms (Chang et al., 2018).

**FIGURE 2:**
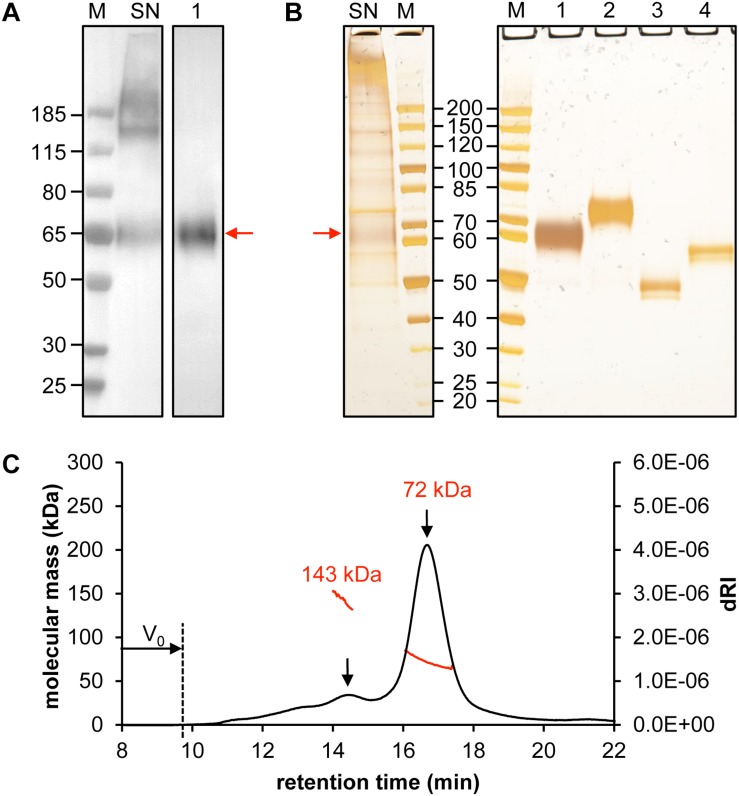
Quality control of purified CD19-AD2. Analysis of 30 μL supernatant of CD19-AD2 expressing CHO-K1 cells and 500 ng SEC purified CD19-AD2 by **(A)** Western Blot detecting His-tagged protein and **(B)** silver-stained SDS-PAGE. Lane M: molecular mass marker; Lane SN: non-reduced supernatant of CD19-AD2 expressing CHO-K1 cells; Lanes 1 to 4: purified CD19-AD2. Lane 1: non-reduced sample; Lane 2: DTT reduced sample; Lane 3: non-reduced PNGaseF digested sample; Lane 4: DTT reduced and PNGaseF digested sample. The monomeric CD19-AD2 band migrating at approximately 60–65 kDa is marked with a red arrow **(C)** SEC-MALS analysis of CD19-AD2. A Superdex 200 10/300 GL column (GE Healthcare, United States) pre-equilibrated with PBS plus 200 mM NaCl (pH 7.4) was loaded with 24 μg of protein upstream to MALS analysis. The molecular weights (kDa) were calculated using the ASTRA software. Both peaks [i.e., dimeric (143 kDa) and monomeric (72 kDa) CD19-AD2 are marked with a black arrow]; representative of two independent experiments are shown.

When we examined the crude supernatant, both monomeric CD19-AD2 as well as high molecular weight aggregates became apparent via anti-His-tagged Western Blot and silver-stained SDS-PAGE (Lane SN, Figures 2A,B). However, following protein purification oligomeric forms could no longer be detected. The apparent molecular weight of CD19-AD2 amounts to 55 kDa when predicted with the use of the ProtParam tool^1^ (Gasteiger et al., 2005) and when considering hydrogen release due to disulfide bond formation and digestion with PNGaseF. Purified and glycosylated CD19-AD2 shows a single band migrating at approximately 60–65 kDa when subjected to SDS-PAGE performed under non-reducing conditions (Lane 1, Figures 2A,B) which shifted to 70–75 kDa when analyzed under reducing conditions (Lane 2, Figure 2B), most likely attributable to a strongly diminished protein compactness after the loss of nine disulfide bonds. PNGaseF-digested CD19-AD2 has a molecular weight of approximately 50 kDa under non-reducing conditions (Lane 3, Figure 2B), which is consistent with the value predicted with the ProtParam tool. Processing the sample under reducing conditions resulted in a band reflective of approximately 60 kDa (Lane 4, Figure 2B). Differences in apparent molecular weight observed between glycosylated and non-glycosylated CD19-AD2 indicate that N-glycosylation of the five putative N-glycosylation sites contributed approximately 15 kDa, i.e., ∼3 kDa per added glycan, as is consistent with previous reports (Croset et al., 2012).

To investigate the prevalence of monomeric or oligomeric content of purified CD19-AD2, SEC-MALS measurements were performed. A symmetric main peak with an estimated molecular weight of 72 kDa was obtained which corresponded to monomeric CD19-AD2 (Figure 2C) next to a second considerably smaller peak reflecting a dimeric version of CD19-AD2 with an associated molecular weight of approximately 143 kDa. Immediately after the void volume (V_0_) a slight increase in refractive index could be detected most likely indicative of a small amount of CD19-AD2 oligomers (Figure 2C).

### CD19-CAR-T Cell Interactions Studies Confirm the Biological Activity of CD19-AD2

**FIGURE 3:**
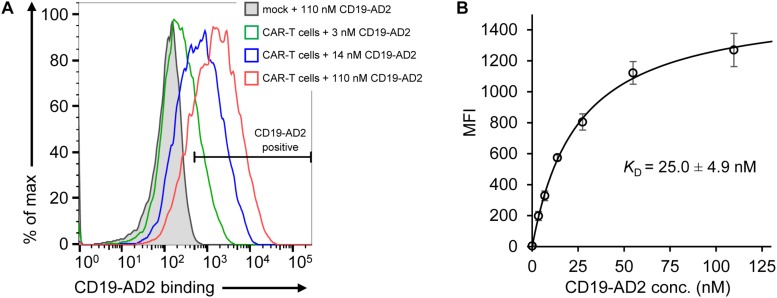
Binding of CD19-AD2 to CD19-directed CAR-T cells. **(A)** CAR-T cells were incubated with various concentrations of CD19-AD2-AF555 (three representative concentrations are shown) and subjected to flow cytometry. The filled gray histogram represents the incubation of mock-T cells with the highest concentration of CD19-AD2-AF555. One experiment representative of four independent experiments is shown. **(B)** CD19-AD2 binding isotherm was generated by fitting the data points obtained from titrating CD19-AD2-AF555 to CAR-T cells to a one-set-of-sites binding model (*K*_D_ = 25.0 ± 4.9 nM, *n* = 4).

After validation of the predominantly monomeric nature of purified CD19-AD2, specific binding was evaluated using CD19-reactive CAR-T cells (CD19-CAR-T cells). The binding moiety of the CAR is composed of the scFv of FMC63 (Nicholson et al., 1997; Minutolo et al., 2019) recognizing a structural epitope on CD19-ECD. As determined by flow cytometry, we observed specific binding of Alexa Fluor 555-labeled CD19-AD2 (CD19-AD2-AF555) to CD19-CAR-T cells in a concentration-dependent manner confirming the structural integrity of CD19-AD2 (Figure 3A): CD19-CAR-transduced T cells incubated with different concentrations of CD19-AD2-AF555 showed a significant shift in fluorescence intensity when compared to non-transduced T cells (Figure 3A). Titrated labeling of CD19-CAR-T cells with CD19-AD2-AF555 allowed us to determine the binding strength between CD19-AD2 and the CD19-CAR. Through fitting the data from flow cytometric analysis to an equation describing a single transition (Hulme and Trevethick, 2010) we arrived at a *K*_D_ of 25.0 ± 4.9 nM (Figure 3B).

**FIGURE 4:**
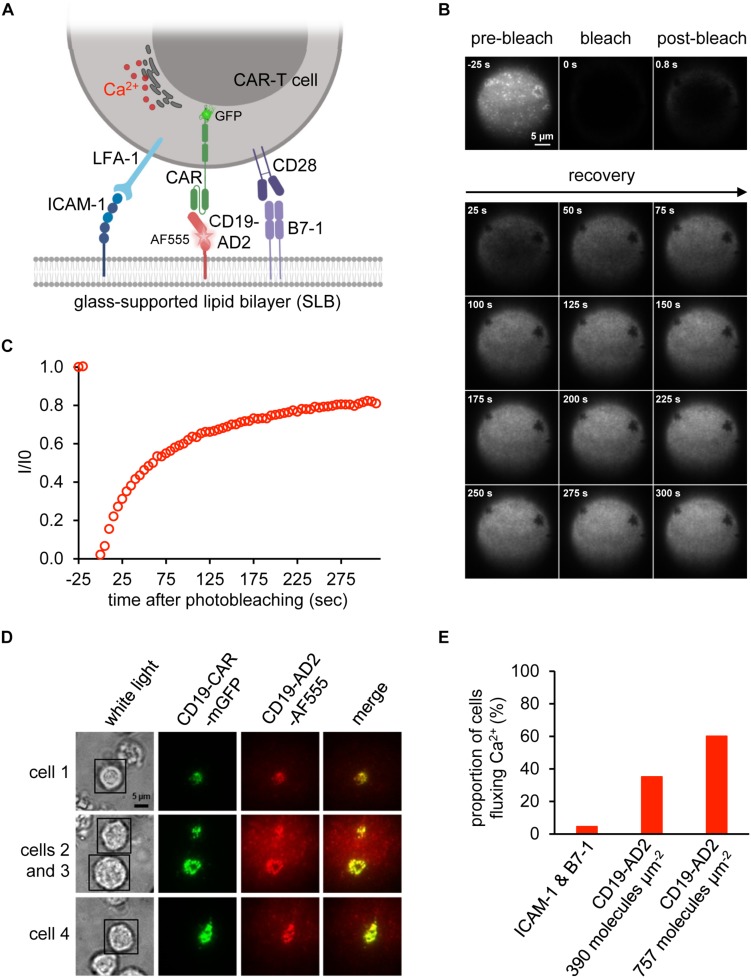
Activation of CAR-T cells. **(A)** Schematic representation of a CD19-CAR-T cell’s immune synapse created with BioRender.com. The SLB was functionalized with the adhesion molecule ICAM-1, the costimulatory molecule B7-1 and CD19-AD2-AF555 for recognition by GFP-tagged CD19-specific CAR-T cells. Upon activation, CAR-T cells release Ca^2+^ from the ER into the cytosol to initiate signaling. **(B)** Fluorescence Recovery After Photobleaching (FRAP) analysis to assess the integrity of the glass-supported planar lipid bilayer (SLB) carrying AF555-labeled CD19-AD2. Images of distinct time points of the experiment until 300 s are shown. **(C)** FRAP quantification of the experiment shown in (A). Values indicate the intensity (I) within the bleached area divided by the initial intensity (I_0_) prior to bleaching. **(D)** Formation of immunological synapses between CD19-AD2 and CD19-CAR-T cells monitored by visualizing CD19-CAR-GFP (shown in green) and CD19-AD2-AF555 (shown in red) using TIRF microscopy. The merge panel (shown in yellow) indicates the successful binding of CD19-CAR-GFP to CD19-AD2-AF555 and formation of an immune synapse. Four representative cells are shown. **(E)** Evaluation of CD19-CAR-T cells fluxing Ca^2+^ for determination of the biological activity of CD19-AD2-AF555. The proportion of Ca^2+^ signaling cells at two different CD19-AD2-AF555 densities on the SLB was measured. As negative control, cells were additionally confronted with antigen-free SLB presenting only ICAM-1 and B7-1.

Biological activity of CD19-AD2 was further confirmed in experiments involving antigen-specific activation of CD19-CAR-T cells and the use of planar glass-SLB, which had been functionalized with CD19-AD2 as well as costimulatory B7-1 and the adhesion molecule ICAM-1 to serve as surrogate for the plasma membrane of a CD19-positive target cell (Figure 4A). Image acquisition was conducted in total internal reflection (TIR) mode to substantially reduce background fluorescence and thereby allow for quantitative microscopy with single molecule resolution (Axelrod et al., 1983; Axmann et al., 2015). Importantly, the use of SLBs as surrogates for target cells in combination with TIRF microscopy is key to mechanistic studies on CAR-T cell performance. Our previous attempts to conduct such experiments had so far been frustrated by recombinant CD19 forming large aggregates prior to bilayer decoration. To ensure best conditions for CD19-CAR-T cell stimulation we assessed the lateral mobility of CD19-AD2-AF555 by performing fluorescence recovery after photobleaching (FRAP) experiments. To monitor fluorescence recovery over time, images were taken prior to and after photo-bleaching (Figure 4B). As shown in Figure 4C, close to 90% fluorescence recovery could be observed within the first 5 min after photobleaching indicating lateral mobility of labeled CD19-AD2 within the SLB (Axmann et al., 2015).

To assess whether CD19-AD2 molecules are efficiently recognized by CD19-CAR-T cells, we incubated CD19-CAR-T cells with SLBs, which had been functionalized with ICAM-1 for LFA-1-mediated adhesion, the costimulatory molecule B7-1 and fluorescence-labeled CD19-AD2 for CAR-mediated stimulation (Figure 4A). As shown in Figure 4D, CD19-CAR-T cells formed mature synapses as witnessed by the rapid emergence of so-termed central Supra-Molecular Activation Clusters (cSMACs) in the center of the contact area. Such synaptic structures are highly enriched in antigen-engaged CARs (Davenport et al., 2018) and result from CARs which have in analogy to their T cell antigen receptor counterparts been previously triggered through ligand engagement in the synaptic periphery to move via active cellular transport mechanisms to the synaptic center (Grakoui et al., 1999; Huppa and Davis, 2003; Joseph et al., 2014). Moreover, as shown in Figure 4E, CD19-CAR-T cells responded specifically and in a density-dependent manner to SLB-anchored CD19-AD2 with a robust rise in intracellular calcium, a second messenger downstream of CAR-proximal signaling as monitored with the use of the ratiometric calcium-indicator fura-2 (Neher, 1995). Taken together, these results testify to the structural integrity and functionality of the recombinantly produced CD19-AD2.

## Discussion

Given its abundance on the surface of newly diagnosed B cell tumors, CD19 has been employed with impressive success rates as a molecular target for CAR-T cell immunotherapy of B cell malignancies, which ultimately resulted in the FDA approval of two CD19-CARs (Pettitt et al., 2018; Hou et al., 2019). However, more recently it has also become evident that up to 50% of treated patients may suffer cancer relapses years after infusion of CD19-CAR-T cells due to the recurrence of B cell tumors. with downregulated CD19 expression or mutated CD19 epitopes not recognized by the CAR (Lim and June, 2017; Majzner et al., 2017). This development clearly underscores the need for CD19-CAR-T cell optimization, which will ultimately require rigorous testing and quantitative approaches with the use of soluble, recombinant CD19. Here, we succeeded in producing considerable amounts of the properly folded extracellular domain of the DTE protein CD19. Critical to efficient expression was the use of (i) the AD2-fusion tag to improve protein solubility, (ii) the BAC as vector backbone for generation of a stable CHO-K1 cell line and (iii) the addition of VPA as chemical chaperone. To further improve CD19-AD2 titers we changed media on a daily basis to maximize producer cell densities for increased secretion of recombinant protein. The short residence time of the product within the bioreactor also contributed to a high and consistent product quality (Bielser et al., 2018). Moreover, media supplementation with the chemical chaperone VPA proved beneficial for the production of CD19-AD2. In transfected CHO cells, VPA has been shown to induce the expression of molecular chaperones which are known to enhance the folding capacity within the secretory pathway and increase in turn productivity (Segar et al., 2017). When testing VPA within a batch process using the generated CD19-AD2 expressing CHO-K1 cell line a 1.7-fold increase in q_P_ was observed when compared to protein production under conditions without supplementation. This could be confirmed within the semi-continuous perfusion experiments with average q_P_ values of 1.4 pg cell^–1^ day^–1^ (addition of VPA) and 0.8 pg cell^–1^ day^–1^ (no chemical chaperones).

In addition to aiming for improved expression yields, the recombinantly produced CD19-AD2 will have to meet certain quality standards with regard to purity, homogeneity as well as conformational stability to support further analyses (Manta et al., 2011; Oliveira and Domingues, 2018). To this end we have paid considerable attention to the molecular design of the recombinant protein. Of note, various studies as well as commercial providers have employed human CD19-ECD encompassing a variety of extracellular domain lengths (De Oliveira et al., 2013; Chang et al., 2018; Teplyakov et al., 2018). In line with the UniProtKB entry (ID: P15391), the CD19-ECD within this study extended from P20 to K291 including 13 residues from exon 5. De Oliveira et al. (2013) produced different CD19-ECD variants encompassing exons 1 to 4 or exons 1 to 3 only. The crystal structure of CD19-ECD (PDB ID: 6AL5, residues E21-R277, Figure 1A) features an intertwined assembly of exons 2 and 4 raising the question as to how a CD19-ECD version missing exon 4 can fold correctly. However, *in vivo* studies provided evidence that an isomer variant of CD19 lacking exon 2 has resulted in resistance against CAR-T cell therapy in patients suffering from ALL (Fischer et al., 2017). Furthermore, given that the X-ray structure of CD19-ECD encompasses exons 1 to 4, the structure of exon 5 remains unresolved (Figure 1A). However, this extracellular part of exon 5 exhibits a high hydrophobic content including amino acids predicted to exert a higher propensity to form helices (Costantini et al., 2006). By using the secondary structure prediction server JPred4, this fragment was estimated to be mostly composed of helical content (Drozdetskiy et al., 2015). The potential short α-helical stretch resembles an extracellular juxtamembrane motif that is important for protein-protein interactions and which has also been reported for other receptors (Sulis et al., 2008; Lu et al., 2010; Lemmon et al., 2014; Shen et al., 2019). It is hence conceivable that the hydrophobic nature of exon 5 also hampers proper protein expression.

To increase solubility of aggregation-prone CD19-ECD, we made use of the AD2 of HSA as a solubility tag. One often employed strategy is to test for each DTE protein a panel of different solubility tags as a means to select the best performing one for the respective DTE protein. Although N-terminal tags are reported to be often advantageous over C-terminal tags, the optimal location of the fusion tags remains a subject of trial and error (Waugh, 2005; Costa et al., 2014). For investigation of immunological synapse formation, AD2 was fused to the C-terminus of CD19-ECD to provide the native N-terminal part of CD19 for recognition by CAR-T cells. Note that while this study was performed, the crystal structure of a glycan mutant of CD19-ECD was resolved featuring a close proximity of C- and N- terminal strands (i.e., 29.4 Å distance between the C^α^ atoms of E21 and R277) (Teplyakov et al., 2018). In the course of this study, we also investigated the expression of the fusion between CD19-ECD and the human crystallizable fragment (Fc) of an IgG1. However, the generated CHO-K1 cell line expressed the Fc fusion construct very poorly and of minor quality (data not shown). Moreover, the Fc fusion strategy would have formed homodimers instead of monomers. But monomeric CD19 is known to associate directly with CD21 and CD81 (Wang et al., 2012) rather than a dimeric CD19 version. The SEC-MALS measurement confirmed that the purified CD19-AD2 is predominantly monomeric and only contains a small fraction of homodimers. However, these homodimers do not arise from misfolded protein or incorrect disulfide bonding but from non-covalent interactions as is indicated by the emergence of a single monomeric band when subjected to non-reducing SDS-PAGE and Western blot analysis.

To assess functional integrity, CD19-AD2 was evaluated within a biological context through recognition and activation of CAR-T cells. Just as the clinically approved CAR-T cell therapies, the CAR-T cells used in this study were equipped with the same CD19-specific murine FMC63-derived scFv (Nicholson et al., 1997; Minutolo et al., 2019). Reports about the binding affinity between CD19 and the monovalent scFv of FMC63 are rare. However, Sommermeyer et al. (2017) titrated a panel of different scFvs including FMC63 to CD19 transduced K562 cells. Following flow cytometric analysis, a binding affinity of 6.1 nM for the scFv of FMC63 was obtained. In another study, flow cytometric binding analysis of the FMC63-based scFv fused to diphtheria toxin to CD19^pos^ JeKo-1 cells resulted in a *K*_D_ value of 47.4 nM (Zheng et al., 2017). A *K*_D_ value of 25.0 ± 4.9 nM obtained for the binding of CD19-AD2 to the FMC63-derived CAR-T cells in our study is within the range and thus in good agreement with the previously reported data. The specific and dose-dependent response of CD19-CAR-T cells to SLB-anchored CD19-AD2 as evaluated by TIRF microscopy of SLB-resident antigen and Ca^2+^ flux analysis further validated proper protein conformation and biological activity.

Taken together, our study illustrates the path from the molecular design to the successful expression of the full-length extracellular domain of the “difficult-to-express” protein CD19. Based on the data presented we conclude that the expressed and purified CD19-AD2 fusion protein is monomeric, highly stable and natively folded as witnessed by its capacity to specifically bind to and efficiently activate CAR-T cells.

## Materials and Methods

### Generation of a Stable CD19-AD2 Expressing CHO-K1 Cell Line

The pL-CD19-AD2 mammalian expression plasmid served as a shuttle vector for integration of the gene of interest (GOI) into the *Rosa26*^BAC^ backbone (Blaas et al., 2007). The GOI was synthesized from GeneArt and composed of the native CD19 signal peptide followed by the coding sequence of the extracellular domain of human CD19 (UniProt ID: P15391, residues P20-K291), a glycine-serine linker (G_4_S)_3_, the domain 2 of HSA (UniProt ID: P02768, residues D211-P413), a GTS linker and a 12x histidine tag (Figure 1A). The whole expression cassette was flanked by two attB target sequences of the integrase φC31 (Belteki et al., 2003; Blaas et al., 2007). The *Rosa26*^BAC CAGGS CD19–AD2^ vector was assembled by homologous recombination using a thermosensitive shuttle vector system as previously described (O’Connor et al., 1989; Guzman et al., 1995; Yang et al., 1997; Zboray et al., 2015). Briefly, electrocompetent *E. coli* DH10B harboring *Rosa26*^BAC^ were transformed with the thermosensitive expression plasmid pRedET (Zhang et al., 1998; Wang et al., 2006). Selection of positive clones was carried out at 30°C. For transformation with the pL-CD19-AD2 shuttle vector, expression of the recombinogenic proteins was induced by addition of L-arabinose (Merck, Germany) and a temperature shift to 37°C. The assembled *Rosa26*^BAC CAGGS CD19–*AD*2^ gene transfer vehicle was purified with the NucleoBond Xtra BAC purification kit (Macherey-Nagel, Germany), linearized with PI-SceI and used for generation of a stable CD19-AD2 production clone from serum-free adapted CHO-K1 (ATCC CCL-61), similarly as previously described (Montigny et al., 2003; Zboray et al., 2015). 1 × 10^6^ CHO-K1 host cells were transfected with 5 μg of linearized *Rosa26*^BAC CAGGS CD19–AD2^ vector complexed with 25 μL lipofectin in CD-DG44 medium (both from Thermo Fisher Scientific, United States). Selection with CD-CHO medium (Thermo Fisher Scientific, United States) containing 4 mM L-glutamine (Carl Roth, Germany), 15 mg L^–1^ phenol-red (Merck, Germany) and 0.5 mg mL^–1^ G418 (Thermo Fisher Scientific, United States) started 24 h post-transfection in one 384-well plate (Corning, United States). Monoclonal cell lines were generated by a second round of subcloning with limiting dilution and screening of culture supernatants with an anti-HSA sandwich ELISA. The best performing clone was chosen for recombinant expression of CD19-AD2 using 50 mL TubeSpin bioreactor tubes (TPP Techno Plastic Products AG, Switzerland) and CD-CHO selection medium containing anti-clumping agent (1:500, Thermo Fisher Scientific, United States). Cells were cultivated at 37°C, 80% humidity, 7% CO_2_ and 220 rpm in a Climo-Shaker ISF1-XC (Adolf Kühner AG, Switzerland). Cell concentrations and viabilities were regularly monitored using a Vi-Cell XR cell counter (Beckman Coulter, United States). The original *Rosa26*^BAC^ clone (RP24–85I15) was obtained from the BAC PAC Resources Children’s Hospital Oakland Research Institute (CHORI).

### Effects of Chemical Chaperones

To improve protein folding and expression titers the addition of three different chemical chaperones was tested based on previous studies [i.e., 2% (v/v) DMSO, 1 mM PBA and 0.5 mM VPA, all from Merck, Germany] (de Almeida et al., 2007; Hwang et al., 2011; Johari et al., 2015; Segar et al., 2017). A total volume of 30 mL medium per bioreactor tube was inoculated with 0.3 × 10^6^ cells mL^–1^ and cultivated in batch mode until day 3. On days 4 and 5 the cell suspension was centrifuged (370 *g*, 10 min) and medium was exchanged. Chaperones dissolved in sterile dH_2_O were added on day 5. On day 7 medium exchange including supplementation of chemical chaperones was repeated. Addition of sterile dH_2_O instead of chemical chaperones served as a negative control. The IVCD and the q_P_ were calculated as described previously and are summarized below (Chusainow et al., 2009; Hennicke et al., 2019),

(1) $$\Delta \,I\,V\,C\,D=\frac{V\,C\,D_{i}-V\,C\,D_{i-1}}{\mu_{\nu }}$$

(2) $$q_{P}=\frac{\Delta \,t\,i\,t\,e\,r}{\Delta \,I\,V\,C\,D}$$

where IVCD is the IVCD in cell day mL^–1^, VCD is the viable cell density in cells mL^–1^, μ_v_ is the viable specific growth rate in day^–1^, q_P_ is the specific productivity in pg cell^–1^ day^–1^ and titer is the product titer in pg mL^–1^. Note, that for semi-perfusion experiments the Δtiter is the apparent titer due to complete removal of the supernatant containing CD19-AD2.

### Anti-HSA Enzyme Linked Immune-Sorbent Assay

To assess the recombinant production of CD19-AD2, quantification was performed by anti-HSA sandwich ELISA (Engvall and Perlmann, 1972). Briefly, 96-well MaxiSorp plates (Thermo Fisher Scientific, United States) were coated with 200 ng per well of the polyclonal goat anti-human albumin antibody (Bethyl Laboratories, Inc., United States) for product capture. Sample dilutions were prepared in PBST with 1% polyvinylpyrrolidone (PVP) (Merck, Germany). Antibody coated MaxiSorp plates were blocked and subsequently incubated with pre-diluted samples. Bound CD19-AD2 was detected with 50 ng per well of the goat anti-human albumin antibody peroxidase conjugate (Bethyl Laboratories, Inc., United States). Plates were developed using 3,3′,5,5′-tetramethylbenzidin stabilized chromogen plus H_2_O_2_ (Merck, Germany). The reaction was stopped by addition of 2.5M H_2_SO_4_ prior to analysis on an Infinite M1000 plate reader (Tecan, Switzerland) at 450 nm. As reference standard serial dilutions (200 to 1.6 ng mL^–1^) of purified CD19-AD2 were used.

### Production of CD19-AD2

To produce adequate amounts of protein for further experiments, CD19-AD2 expressing CHO-K1 cells were cultivated in bioreactor tubes in a total volume of 10 mL inoculated with 0.3 × 10^6^ cells mL^–1^. After 3 days of batch cultivation the selection medium was changed every day until day 13 to increase cell concentration and to harvest high volumes of culture supernatant. From day 5 on the medium was supplemented with 0.5 mM VPA from a 1000x stock solution. For comparison, the same experiment was performed without addition of VPA. Supernatants were stored at 4°C.

### Bio-Layer Interferometry (BLI) Measurements

CD19-AD2 titers of the semi-continuous perfusion experiments were determined using a bio-layer interferometry assay on an Octet RED96e (ForteBio, United States). The entire experiment was performed at 25°C using Streptavidin (SA) biosensors (ForteBio, United States) with the plate shaking at 1000 rpm. Prior to sample preparation, the culture supernatants were centrifuged at 16000 *g* for 10 min. SA biosensors were equilibrated in neutralization solution (NS) [PBS buffer containing 0.05% (v/v) Tween20 and 1% BSA] and then loaded for 300 s with 5 μg mL^–1^ biotinylated anti-human CD19 mAb (clone HIB19; BioLegend, United States) diluted in the same buffer. To record a baseline, the biosensors were dipped in a mixture of NS solution and CHO-K1 mock supernatant within a ratio of 1:2 for 60 s. This step was repeated for 180 s to obtain a stable baseline, before the biosensors were submerged for 60 s into a 1:2 mixture of NS solution and sample supernatant for capturing of CD19-AD2. SA biosensors were regenerated in regeneration solution (RS) (0.1M glycine buffer, pH 2.5) and NS solution according to the manufacturer’s instructions. The standard curve was prepared by spiking of purified CD19-AD2 in varying concentrations (30–0.94 μg mL^–1^) into the 1:2 mixture of NS solution and CHO-K1 mock supernatant. Each experiment included a baseline measurement (negative control) as well as one point of the standard curve with known concentration (positive control). Note, no unspecific binding to CHO-K1 mock supernatant was observed. Additionally, data were evaluated under consideration of the limit of detection (LOD) and limit of quantification (LOQ) as reported elsewhere (Armbruster and Pry, 2008; Carvalho et al., 2018). Analysis was performed using the Octet data analysis software version 11.1.1.39 (ForteBio, United States) according to the manufacturer’s guidelines.

### Purification of CD19-AD2

Pooled cell culture harvests were centrifuged (4800 *g*, 15 min), filtered (0.45 μM Supor^®^ membrane filter, Pall Corporation, United States), 20-fold concentrated and diafiltered with 20 mM phosphate buffer containing 500 mM NaCl and 40 mM imidazole (pH 7.4) using a Labscale TFF System equipped with a 30 kDa cut-off Pellicon^TM^ XL device (Merck, Germany). The ÄKTA pure chromatography system (GE Healthcare, United States) was used for the following purification steps. The diafiltrated solution was loaded onto a 1 mL HisTrap HP column (GE Healthcare, United States) and bound protein eluted applying a linear gradient of 40 to 500 mM imidazole over 20 column volumes. Fractions containing CD19-AD2 were pooled and dialyzed against 20 mM phosphate buffer containing 200 mM NaCl (pH 7.4) using a Spectra/Por Dialysis tubing (Spectrum Chemical Mfg. Corp., United States) at 4°C overnight. Dialyzed protein was concentrated using Amicon Ultrafilters with a cut-off of 10K (Merck, Germany) and subjected to SEC on a HiLoad 16/600 Superdex 200 pg column (GE Healthcare, United States) equilibrated with the same buffer used for dialysis. Fractions containing only monomeric CD19-AD2 were pooled, concentrated and stored at −80°C for all further analysis steps.

### Protein Analysis for Purity and Quality

#### SDS-PAGE and Western Blot Analysis

The SDS-PAGE was carried out using Bolt 4–12% Bis-Tris Plus Gels and NuPAGE MOPS SDS Running Buffer (all from Thermo Fisher Scientific, United States). To remove N-glycosylations, purified samples were incubated with PNGaseF (Roche, Switzerland) according to the manufacturer’s instructions. A total volume of 30 μL of pooled supernatants and 500 ng of purified or deglycosylated CD19-AD2 were mixed with NuPAGE LDS Sample buffer (4x), heated to 70°C for 10 min and loaded onto the gel. For reducing conditions purified samples were mixed with 10x NuPAGE sample reducing agent containing DTT and heated to 95°C for 5 min. The protein bands of the gels were subjected to silver staining using glutaraldehyde as a sensitizer as previously described (Wray et al., 1981; Chevallet et al., 2006). For Western Blot analysis, the proteins were electrotransferred onto a PVDF membrane (Carl Roth, Germany). After blocking, the membrane was incubated with an anti 6x-His tag biotinylated monoclonal antibody (Thermo Fisher Scientific, United States), which was detected with a streptavidin-HRP conjugate (GE Healthcare, United States). For visualization of protein bands, the membrane was incubated with SuperSignal West Pico PLUS Chemiluminescent Substrate (Thermo Fisher Scientific, United States) and subjected to the FUSION-FX7 Spectra chemiluminescence imaging system (Vilber, France). The PageRuler Unstained and Prestained Protein Ladders (both from Thermo Fisher Scientific, United States) were used as a size marker for SDS-PAGE and Western Blot, respectively.

#### Size-Exclusion Chromatography-Multi Angle Light Scattering (SEC-MALS)

Size-exclusion chromatography combined with multi-angle light scattering (SEC-MALS) was used to determine the purity, the aggregation behavior and the molar mass of CD19-AD2. Analysis was performed on an LC20 prominence HPLC system equipped with the refractive index detector RID-10A, the photodiode array detector SPD-M20A (all from Shimadzu, Japan) and a MALS Heleos Dawn8C plus QELS detector (Wyatt Technology, United States). A Superdex 200 10/300 GL column (GE Healthcare, United States) was used and equilibrated with PBS containing 200 mM NaCl (pH 7.4) as running buffer. Experiments were performed at a flow rate of 0.75 mL min^–1^ at 25°C and analyzed using the ASTRA 6 software (Wyatt Technology, United States). Determination of the molar mass of a sample of bovine serum albumin was performed to verify proper performance of MALS. Prior to analysis, samples were thawed, centrifuged (16000 *g*, 10 min) and filtered using 0.1 μm Ultrafree-MC filter (Merck Millipore, Germany). A total amount of 24 μg CD19-AD2 was injected for each measurement.

### Analysis for Specific Binding to CAR-T Cells

#### Generation and Cultivation of CAR-T Cells

Buffy coats from anonymous healthy donor’s blood were purchased from the Austrian Red Cross, Vienna. CD3^+^ primary human T cells were isolated using the RosetteSep Human T cell Enrichment Cocktail (STEMCELL Technologies, Canada) and immediately cryopreserved in RPMI-1640 GlutaMAX medium (Thermo Fisher Scientific, United States) supplemented with 20% FCS and 10% DMSO (both from Merck, Germany). Primary human T cells were thawed in RPMI-1640 GlutaMAX medium, supplemented with 10% FCS, 1% penicillin-streptomycin (Thermo Fisher Scientific, United States) and 200 IU mL^–1^ recombinant human IL-2 (Peprotech, United States) and activated with Dynabeads Human T-Activator CD3/CD28 beads (Thermo Fisher Scientific, United States) at a 1:1 ratio according to the manufacturer’s instructions. 24 h after stimulation, T cells were transduced in cell culture plates, which were coated with RetroNectin (Takara, Japan), according to the manufacturer’s instructions. Thawed lentiviral supernatant was added to the T cells at a final dilution of 1:2, yielding a cell concentration of 0.5 × 10^6^ cell mL^–1^. 48 h after transduction, selection of CAR-T cells was performed by treatment with 1 μg mL^–1^ puromycin (Merck, Germany) for 2 days. Transduced T cells were cultivated in AIMV medium (Thermo Fisher Scientific, United States) supplemented with 2% Octaplas (Octapharma, Switzerland), 1% L-glutamine, 2.5% HEPES (both from Thermo Fisher Scientific, United States) and 200 IU mL^–1^ recombinant human IL-2.

#### Construction of Lentiviral Vector

VSV-G pseudotyped lentivirus was generated by co-transfection of Lenti-X 293T cells (Takara, Japan) with a puromycin-selectable pCDH expression vector (System Biosciences, United States) encoding the second-generation anti CD19-CAR (FMC63.4-1BB.ζ) and viral packaging plasmids pMD2.G and psPAX2 (Addgene plasmids #12259 and #12260, respectively; gifts from Didier Trono) using the PureFection Transfection Reagent (System Biosciences, United States) according to the manufacturer’s instructions. Viral supernatants were collected on days 2 and 3 after transfection and were concentrated 100-fold using the Lenti-X Concentrator (Takara, Japan) according to the manufacturer’s instructions. Viral suspensions were frozen at −80°C until further use.

#### Flow Cytometric Analysis of Binding of CD19-AD2 to CD19-Directed CAR-T Cells

1 × 10^5^ CAR-T cells were taken for flow cytometric analysis. Cells were washed once in FACS buffer composed of PBS supplemented with 0.2% human albumin (CSL Behring, United States) and 0.02% sodium azide (Merck, Germany) and subsequently incubated with varying concentrations (3–110 nM) of AF555-labeled (Thermo Fisher Scientific, United States) and SEC purified CD19-AD2 for 25 min at 4°C. Cells were washed twice with ice-cold FACS buffer and data were acquired on an LSR Fortessa instrument (BD Biosciences, United States) and analyzed using the FlowJo software (FlowJo, LLC, United States). Donor-matched non-transduced cells were used as a negative control. T cells from three different donors were analyzed in two independent experiments.

### Activation of CAR-T Cells by CD19-AD2

#### Preparation of Glass-Supported Lipid Bilayers (SLBs)

Supported lipid bilayers were prepared as described by Axmann et al. (2015). In brief, 1,2-dioleoyl-*sn*-glycero-3-[(*N*(5-amino-1-carboxypentyl)iminodiacetic acid) succinyl] [nickelsalt] (Ni-DOGS-NTA) and 1-palmitoyl-2-oleoyl-*sn*-glycero-3-phosphocholine (POPC) (both from Avanti Polar Lipids, United States) were dissolved in chloroform and mixed in a 1:9 molar ratio. After drying under vacuum in a desiccator overnight, the lipids were resuspended in 10 mL PBS and sonicated under nitrogen at 120–170 W for up to 90 min until the suspension had lost most of its turbidity. To pellet non-unilamellar vesicles, the suspension was centrifuged for 4 h at 55000 *g* at 25°C. The clear supernatant was centrifuged again for 8 h at 74000 *g* and 4°C, filtered through a 0.2 μm syringe filter (Sarstedt, Germany) and stored under nitrogen at 4°C for up to 6 months. Glass slides (24 mm × 50 mm #1 borosilicate, VWR, United States) were immersed in a 2:1 mixture (v/v) of concentrated sulfuric acid and 30% hydrogen peroxide (both from Merck, Germany) for 60 min, rinsed with deionized H_2_O and air-dried. Cleaned slides were attached to the bottom of an 8-well Lab-Tek^TM^ chamber (Thermo Fisher Scientific, United States) with picodent twinsil extrahart (Picodent, Germany) until the glue had solidified. The lipid vesicle suspension was diluted 1:20 in PBS, filtered through a 0.2 μm filter and split in 200 μL portions per well to form a contiguous SLB. Chambers were washed twice with 15 mL PBS to remove excessive vesicles. 12x His-tagged proteins of interest were added to the SLBs and incubated for 60 min in the dark at room temperature. Finally, chambers were rinsed twice with 15 ml PBS to remove unbound protein.

Fluorescence recovery after photobleaching -based experiments were conducted as published (Axmann et al., 2015) to determine the fraction of immobile SLB-resident molecules. In brief, a circular aperture was placed in the excitation beam path. After recording 5 images, the SLB was subjected to an intense bleach pulse, which was followed by 1 image every 5 s acquired for 320 s. Intensity values of the acquired images were normalized against intensities measured in images acquired prior to bleaching.

#### Single Molecule Fluorescence Microscopy

Microscopy was conducted with two inverted setups, which had been custom-built to allow for calcium and TIR-based imaging. Excitation light was provided by diode lasers featuring 488, 647 (both iBeam smart Toptica), and 532 nm (OBIS) laser lines on both microscopy setups, which were also equipped with a custom Notch filter (Chroma Technology) to block reflected stray light of 488, 532, or 640 nm from reaching the camera.

(i) An inverted microscope (DMI 4000, Leica) was equipped with a chromatically corrected 100x TIR objective (HC PL APO 100x/1.47 OIL CORR TIRF, Leica), a 20x objective (HC PL FLUOTAR 20x/0.50 PH2 ∞/0.17/D, Leica) and in addition to the lasers specified above with a mercury lamp (EL6000, Leica) for Fura-2-based calcium recordings. Furthermore, this microscope included two fast-filter wheels containing 340/26 and 387/11 excitation bandpass filters (both Leica), and ET525/36, ET605/52 and ET705/72 emission bandpass filters present in the emission pathway (all Leica), as well as a motorized multichroic mirror turret equipped with FU2 (Leica) and ZT405/488/532/647rpc beam splitters (AHF).
(ii) A second inverted microscope (Eclipse Ti-E, Nikon) was equipped with a chromatically corrected 100x TIR objective (CFI SR Apo TIR 100x Oil NA:1.49, Nikon), a 20x objective (CFI S Fluor 20x NA:0.75, Nikon) and, in addition to specified lasers, a xenon lamp (Lambda LS, Sutter Instrument) providing illumination for Fura-2-based calcium measurements. This microscope featured also a Lambda 10-3 excitation filter wheel (Sutter Instrument) equipped with 340/26 and 387/11 bandpass filters (both AHF), and a Lambda 10-3 emission filter wheel featuring bandpass filters ET510/20, ET605/52, ET700/75 (all AHF). In addition, two polychroic filter turrets allowed for the implementation of two independent beam paths for laser- and lamp-mediated illumination involving zt405/488/532/640rpc and 409/493/573/652 beam splitters (both AHF).

Data were recorded using an iXon Ultra 897 EM-CCD camera (Andor). An 8 channel DAQ-board PCI-DDA08/16 (National Instruments) and the microscopy automation and image analysis software Metamorph (Molecular Devices) were used to program and apply timing protocols and control all hardware components. Data analysis was performed with Fiji image processing package based on ImageJ (Schindelin et al., 2012, 2015). Prism 5 (GraphPad, United States) was used for statistical data analysis and plotting graphs.

#### Monitoring Synaptic Recruitment of CD19-AD2 and CAR-T Cells

CD19-AD2 density measurements were performed exactly as described previously (Axmann et al., 2015). Images were recorded in TIR-mode using the chromatically corrected 100x objective with high numerical aperture (CFI SR Apo TIR 100x Oil NA:1.49, Nikon). Two to ten minutes after seeding, T cells were imaged to monitor (i) the redistribution of SLB-resident CD19-AD2 linked to fluorophore AF555 (using 532 nm laser illumination) and anti CD19 CAR-GFP (using 488 nm laser illumination) appearing in the TIR-sensitive field of evanescent light. Changes in intracellular calcium levels were measured via the ratiometric calcium-sensitive dye Fura-2-AM (Thermo Fisher Scientific) as described (Roe et al., 1990). Therefore, 1 × 10^6^ CAR-T cells were incubated in 1 mL conditioned medium supplemented with 5 μM Fura-2-AM for 30 min at room temperature, washed twice in 1 mL ice-cold imaging buffer (HBSS supplemented with 2 mM CaCl_2_, 2 mM MgCl_2_, 2% FCS, and 10 mM HEPES), resuspended in 50 μL imaging buffer and stored on ice for up to 2 h. For calcium imaging, CAR-T cells were placed in close proximity to the SLB. As soon as the first cells touched the SLB, 510/80 nm emission was recorded with 340 and 387 nm excitation every minute for 15 min, visiting five different XY-stage positions and using a UV-transmissive 20x objective (CFI S Fluor 20x NA:0.75, Nikon, Japan). For analysis, 340 nm and 387 nm images were subjected to rolling ball background subtraction (Fiji, built-in plugin). Resulting 340 nm stacks were divided by the corresponding 387 nm stacks to generate ratiometric stacks. Individual cells were detected in the 340 nm channel by using Difference of Gaussians detector (Lowe, 2004), tracked in XY and time by using Linear Assignment Problem tracking (Jaqaman et al., 2008) with the use of the Fiji Trackmate plug-in Tinevez et al. (2017). XY-plane positions of the detected cells and their corresponding tracks were then applied to ratiometric stack and integrated fluorescence intensity values pertaining to all tracks were recorded. Parameters (i.e., radius of cell, length, and quality of track) were chosen heuristically. Data corresponding to tracked cells were analyzed applying a custom-written python-3 code and python packages (numpy, matplolib, pandas). Intensity values corresponding to individual tracks were smoothened with a Gaussian rolling window filter, whereupon we identified a frame in which the change in intensity was maximal. Upon identifying this frame of activation, the raw data corresponding to the entire time series were normalized to an average intensity value of all frames prior to the frame of activation. After normalization, tracks were classified as activating when the average intensity value of three consecutive frames (i.e., spanning 3 min) after the frame of activation was > 1.5. The results of each individual track were then combined to characterize the population.

## Data Availability Statement

The datasets generated for this study are available on request to the corresponding author.

## Author Contributions

RK, JH, EL, PM, and VG: conceptualization. EL, AW, VG, BS, and PM: investigation. RK, EL, AW, VG, JH, and BS: data analysis and interpretation. EL, AW, RK, BS, and VG: writing – original draft. EL, RK, AW, JH, VG, PM, BS, and ML: writing – review and editing. RK, JH, EL, VG, and ML: supervision. RK, JH, and ML: funding acquisition.

## Conflict of Interest

The authors declare that the research was conducted in the absence of any commercial or financial relationships that could be construed as a potential conflict of interest.

## Footnotes

1 https://web.expasy.org/protparam/
